# From spatial ecology to spatial epidemiology: modeling spatial distributions of different cancer types with principal coordinates of neighbor matrices

**DOI:** 10.1186/1742-7622-11-11

**Published:** 2014-08-08

**Authors:** Ari Voutilainen, Anna-Maija Tolppanen, Katri Vehviläinen-Julkunen, Paula R Sherwood

**Affiliations:** 1 Department of Nursing Science, University of Eastern Finland, P.O. Box 1627, 70211 Kuopio, Finland; 2 Department of Biology, University of Eastern Finland, Kuopio, Finland; 3 Research Centre for Comparative Effectiveness and Patient Safety, University of Eastern Finland, Kuopio, Finland; 4 School of Pharmacy, University of Eastern Finland, Kuopio, Finland; 5 Department of Acute and Tertiary Care, School of Nursing, University of Pittsburgh, Pittsburgh, PA, USA

**Keywords:** Cancer incidence, Finland, Principal coordinates of neighbor matrices, Spatial epidemiology

## Abstract

**Background:**

Epidemiology and ecology share many fundamental research questions. Here we describe how principal coordinates of neighbor matrices (PCNM), a method from spatial ecology, can be applied to spatial epidemiology. PCNM is based on geographical distances among sites and can be applied to any set of sites providing a good coverage of a study area. In the present study, PCNM eigenvectors corresponding to positive autocorrelation were used as explanatory variables in linear regressions to model incidences of eight most common cancer types in Finnish municipalities (*n* = 320). The dataset was provided by the Finnish Cancer Registry and it included altogether 615,839 cases between 1953 and 2010.

**Results:**

PCNM resulted in 165 vectors with a positive eigenvalue. The first PCNM vector corresponded to the wavelength of hundreds of kilometers as it contrasted two main subareas so that municipalities located in southwestern Finland had the highest positive site scores and those located in midwestern Finland had the highest negative scores in that vector. Correspondingly, the 165^th^ PCNM vector indicated variation mainly between the two small municipalities located in South Finland. The vectors explained 13 - 58% of the spatial variation in cancer incidences. The number of outliers having standardized residual > |3| was very low, one to six per model, and even lower, zero to two per model, according to Chauvenet’s criterion. The spatial variation of prostate cancer was best captured (adjusted *r*^2^ = 0.579).

**Conclusions:**

PCNM can act as a complementary method to causal modeling to achieve a better understanding of the spatial structure of both the response and explanatory variables, and to assess the spatial importance of unmeasured explanatory factors. PCNM vectors can be used as proxies for demographics and causative agents to deal with autocorrelation, multicollinearity, and confounding variables. PCNM may help to extend spatial epidemiology to areas with limited availability of registers, improve cost-effectiveness, and aid in identifying unknown causative agents, and predict future trends in disease distributions and incidences. A large advantage of using PCNM is that it can create statistically valid reflectors of real predictors for disease incidence models with only little resources and background information.

## Background

Epidemiology and ecology share many fundamental research questions: why are the spatiotemporal distributions of diseases/species not uniform? What causes changes in the disease incidence/prevalence or species abundance? How can these changes be predicted? This common ground has generated a rapidly growing discipline, spatial epidemiology [1]. Although spatial epidemiology has made great progress in answering these questions [2], certain methodological problems remained unresolved. For example, including multiple explanatory variables in epidemiological models often leads to multicollinearity [3], and adjusting for potential confounders may create an artificial connection between the outcome and exposures [4] causing both statistical and theoretical challenges. The purpose of this article is to describe and demonstrate how principal coordinates of neighbor matrices (PCNM), a method from spatial ecology, can be applied to spatial epidemiology to address these methodological problems.

PCNM is based on geographical distances between different sites and “can be applied to any set of sites providing a good coverage of a geographical sampling area” [5]. PCNM variables model spatial relationships among sites in decreasing order of spatial scale. Originally, PCNM was developed to deal with induced spatial dependence between statistical units due to effects of external processes. “True” spatial autocorrelation [5,6] refers to a situation when the response variable depends on itself due to internal processes. The PCNM approach is rather simple: if spatial dependence cannot be avoided, it has to be considered as a source of information. PCNM searches for eigenvectors corresponding to positive autocorrelation and uses them to describe spatial structures in a given dataset [5,6].

The PCNM approach is closely related to Moran’s *I*, an index of spatial autocorrelation [7]. Moran’s *I* is a correlation coefficient and it gives a linear relationships (autocorrelation) between dependent variables with respect to a spatial weighting matrix. There are several criteria to create the spatial weights [8]. PCNM can be seen as a particular case of Moran’s eigenvector maps where the spatial weighting matrix is defined with geographical (Euclidean) distances between study locations [6].

The PCNM eigenvectors, reflecting spatial scales in a given dataset are rotated so that they do not correlate with each other and sample locations can be randomly or systematically assigned. Consequently, PCNM statistically avoids the multicollinearity problem. PCNM vectors can be considered as reflections of real predictors if 1) each explanatory variable obeys one or more spatial patterns and 2) the geographic coverage of the sampling area is complete enough so that PCNM reflects most spatial patterns. Therefore, it is possible to create statistically valid models of disease prevalence and incidence by using PCNM vectors as explanatory variables instead of more explicit explanators of diseases, which in some cases can be extremely difficult to measure. PCNM variables act as proxies for any kind of process resulting in spatial structuration of the response variable(s). When evaluating the quality of spatial patterns, it is essential to consider the sampling area’s geographical coverage based on the study sites. Importantly, good coverage does not refer to the size of the area, and thus PCNM can be applied to all spatial scales.

PCNM has several advantages over autoregressive methods which handle autocorrelation by removing the spatial dependency between observations. In the case of disease incidence, PCNM enables structuring statistically reliable models of spatial variation in the incidence regarding the site *i* on the basis of its correspondences with other sites, as the spatial patterns of the incidence, as well as those of factors affecting the incidence, are all included in and explained by the vectors provided by PCNM [9]. The vectors produced by PCNM reflect cyclic variation in the outcome variable so that the first PCNM eigenvector corresponds to the broadest spatial scale indicating the spatial extent of the entire study area (large-scale variation) and the last PCNM eigenvector corresponds to the finest spatial scale (small-scale variation). The PCNM vectors can be applied directly as explanatory variables to regression and canonical models [5,9].

The aim of this study is to present PCNM as a complementary technique, not the method of choice, to the most common and best validated methods for disease mapping, such as the Besag, York, and Mollie model [10] and Poisson-Gamma model(s) [11]. It is proposed that PCNM vectors can be used as proxies for demographics and causative agents to deal with autocorrelation, multicollinearity, and confounding variables. PCNM may also provide *a priori* information for techniques based on Bayesian statistics.

## Methods

### Data

Our example dataset consisted of eight most common cancer types in all Finnish municipalities (*n* = 320) between 1953 and 2010. Cancer incidences were as follows: breast (age-adjusted incidence rate per 100,000 women was 96.6), prostate (men, 85.6), lung including trachea (men, 28.5; women, 12.7), colon (men, 16.1; women, 12.7), skin melanoma (men, 13.9; women, 13.8), rectal (men, 11.6; women, 6.9), stomach (men, 6.8; women, 3.9), and leukemia (men, 6.9; women, 5.9). Different cancer types were dealt with separately because different cancers are different diseases and, therefore, it was highly expectable that they will at least partly follow different spatial patterns [12].

The main reason for the use of a large long-term dataset was to enable inclusion also the smallest municipalities in the analysis. In Finland, there are municipalities with so small population that new cancer cases are not found every year. The effect of “zero years” on the analysis was diminished by pooling the data over as many years as possible and then using the number of new cases per person-year as the unit of incidence. The high number of Finnish municipalities together with their small sizes (median, 425; range, 6 - 15,053 km^2^) provides an excellent coverage of the entire geographical area. The dataset contains no missing values.

The dataset was provided and the research approved by the Finnish Cancer Registry (FCR), which maintains a continuously updated nation-wide database of the incidence of all primary cancers in Finland. Final coding is done by qualified secretaries and supervised by the Registry physician. Reporting of cancers is mandated by law and each cancer considered to be an independent new primary lesion is registered separately and all independent cancer processes are coded as separate entities. FCR collects data with very high accuracy and completeness [13]. Rare cancer types were excluded from the present study to prevent person recognition and because rare events typically create nonlinear relationships between variables.

Data for the study included the number of new cancer cases per year per municipality together with information concerning the sex structure of populations. The unadjusted cancer incidence rate (cases per 1,000 person-years in general, per 1,000 women-years for breast cancer, and per 1,000 man-years for prostate cancer) instead of age-adjusted incidence rate was used as the dependent variable due to the methodological purposes. We expressly wanted to test how well spatial vectors alone explain the variation in disease incidences - without information concerning demographics and possible causative agents. This was important for two reasons: 1) many real predictors of diseases are correlated with each other and 2) information about predictors may not be available. As both multicollinearity and the lack of information decrease the explanatory power of traditional statistical models, PCNM can be performed to deal with these challenges.

When applied to clinical purposes, PCNM vectors can be associated with demographics and possible causative agents of the target variable to search for reasons for the spatial patterns detected. This is simply done by modeling demographics and causative agents by the same PCNM vectors which best explained the spatial distribution of the target variable. In the present study, this was not done as the aim was to test the PCNM method first time in an epidemiologic context, not to find reasons for the spatial distribution of cancer in Finland. Furthermore, information concerning demographics and many supposed causative agents of cancer are available in Finnish Registries mainly from 1980s and 1990s, not from 1950s as cancer incidences. Consequently, a comprehensive comparison between spatial patterns of cancer and those of demographics and causative agents in Finland has to be carried out using a shorter-term dataset than in this study. This could be the next step in introducing the PCNM method to spatial epidemiology.

If variables related to chronic diseases are explained by spatial patterns, it has to be taken into account that the study site may not be the site where the person in question lived when the disease started. This is important especially at individual-level data, but may also affect results at group level if migration waves have been intense and/or long-lasting. From this point of view, Finland provides a good study area, as the propensity for intermunicipal migration in Finland is rather low [14] and two-thirds of Finnish people live in the county where they were born [15].

### Spatial vectors

A principal coordinate analysis of a truncated matrix of Euclidean distances between the 320 Finnish municipalities (as of January 1, 2013) was carried out to create the patterns of spatial scales. First, a 2-dimensional matrix of Euclidean distances (**D**) among the municipalities was conducted using the latitudes and longitudes of geographical centers of settlement concentrations, such as cities, of the municipalities as initial values. The latitudes and longitudes were defined as meters from the equator and meters from the meridian 27° east of Greenwich, respectively. If the municipality had more than one settlement concentration, an average of geographical centers of these concentrations weighed by the number of inhabitants per concentration was calculated and considered as the initial value. The geographical locations were determined by the National Land Survey of Finland. Second, a truncated connectivity matrix (**W**) was constructed according to the following rule: *w*_*ij*_ = *d*_*ij*_ if *d*_*ij*_ = ≤ *t* and *w*_*ij*_ = 4*t* if *d*_*ij*_ > *t*, where *t* is a threshold value indicating the maximum distance i.e. minimum spanning tree which maintains all sites (i.e. municipalities) being connected [5,6]. Third, eigenvectors were extracted from the centered **W**. The PCNM results in vectors corresponding to positive and negative eigenvectors but only the positive ones are taken into account in further analyses. A good reconstruction of spatial structures is obtained by this method [5]. Negative eigenvectors model negative spatial correlation and they may be useful in some instances, but not in the present context. The PCNM vectors were created using functions of the “spacemakeR” package [6] for the R statistical language [16].

### Regression models

Multiple linear regressions with forward stepwise procedure were performed on the cancer incidences to test the explanatory power of the 165 PCNM vectors. Each regression model included one outcome variable (incidence of certain cancer type) and multiple explanatory variables (PCNM vectors). Normality of incidence rate distributions and residuals of the final models were assessed with the Kolmogorov-Smirnov test. If the distribution was non-normal (prostate cancer, skin melanoma, stomach cancer, and leukemia) the incidence rates were log_10_-transformed before linear regression analyses. The number of independent variables, which were able to improve the models more than expected by chance, was approximated on the basis of the adjusted coefficient of determination *r*^2^[17]. In practice, this meant that explanatory variables i.e. PCNM vectors with *p*-value <0.05 (corresponding to the alpha level stopping criterion) were added to the model as long as the adjusted *r*^2^ obtained using all the PCNM vectors as explanatory variables was reached. The “double stopping criterion” was necessary since using PCNM vectors as independent variables in a regression model may result in an inflated *r*^2^ due to the fact that many vectors can reflect the same spatial process [17,18]. The relative significance of single spatial vectors in explaining the cancer incidences was determined on the basis of standardized coefficients. Residual plots were drawn to evaluate the goodness-of-fit in the models. The regression analyses were performed with the IBM SPSS 19 for Windows.

When applying the method to clinical purposes, it is highly important to associate the PCMN vectors also to actual explanatory variables before drawing conclusions, as the spatial processes causing the structures can be represented by one or several PCNM vectors. In general, spatial processes should be modeled by a group of PCNM vectors, not by one vector at a time.

## Results

### PCNM vectors

**Figure 1:**
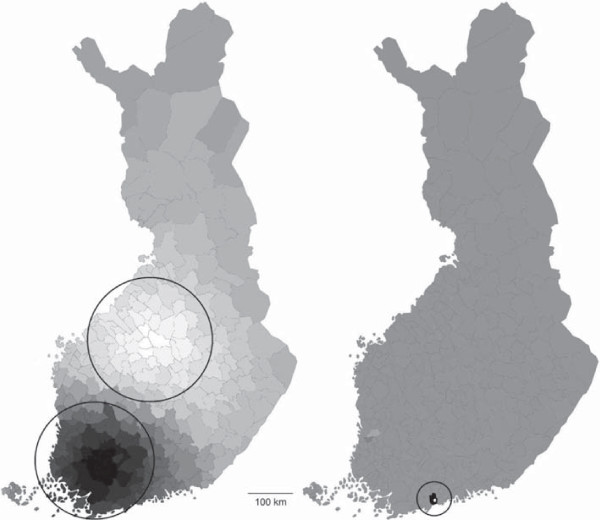
**PCNM patterns corresponding to the largest (eigenvector 1 on the left) and finest spatial scale (eigenvector 165 on the right) in the given data (320 municipalities in Finland).** Dark color indicates high positive site score and light color high negative score in the vector.

PCNM resulted in 165 vectors with a positive eigenvalue. The threshold value *t* was 1,409 meters. It denotes the cycle wavelength of the PCNM vector which reflects the smallest-scale variation in the study area. Shorter wavelengths are possible only if more sample locations are offered to PCNM. As municipalities are irregularly arranged throughout Finland, it was impossible to determine the exact cycle wavelength (spatial scale) of each PCNM vector, in the present case. In practice, however, the first PCNM vector corresponded to the wavelength of hundreds of kilometers as it contrasted two main subareas so that municipalities located in southwestern Finland had the most positive site scores and those located in midwestern Finland had the most negative scores in that vector. In Figure 1, the interpretation concerns the contrast between the dark and light areas. Correspondingly, the 165^th^ PCNM vector corresponded to *t* as it indicated variation mainly between the two small municipalities located in South Finland, Espoo (black area in Figure 1) and Kauniainen (white area in Figure 1) which is actually surrounded by Espoo. PCNM vectors can be regarded as representatives of cyclic variations and actual site scores as “correlation coefficients” between the variations and sample locations. A high absolute score value refers to a high correlation and a low absolute value to a low correlation between the variation and location in question.

### Regression models

**Figure 2:**
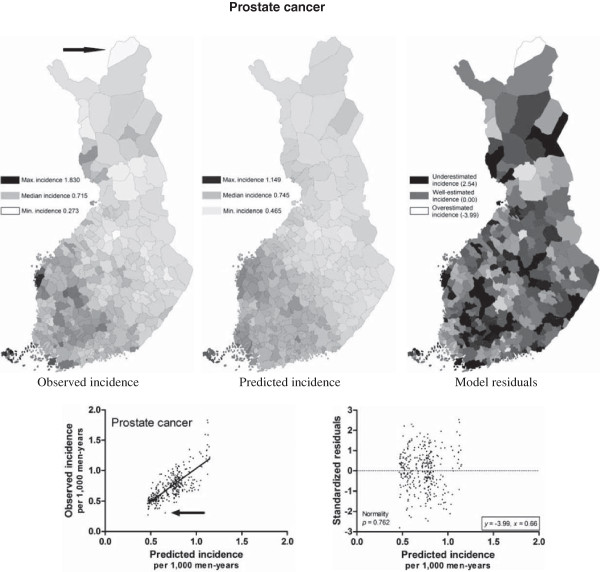
**Observed and modeled incidences of prostate cancer (per 1,000 man-years) expressed in relation to observed minimum and maximum.** Standardized residuals reflect the goodness-of-fit of the model in different areas. The arrow points the outlier.

**Figure 3:**
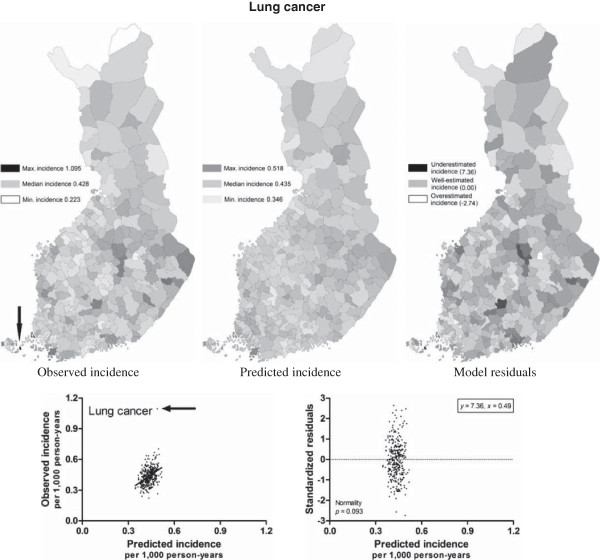
**Observed and modeled incidences of lung cancer (per 1,000 person-years) expressed in relation to observed minimum and maximum.** Standardized residuals reflect the goodness-of-fit of the model in different areas. The arrow points the outlier.

The PCNM vectors explained 13 - 58% of the spatial variation in cancer incidences according to the adjusted *r*^2^ with the double stopping criterion (Table 1). The number of outliers with standardized residual > |3| was 1 - 6 per model, which can be considered low as n in each case was 320. The number of outliers was even lower, 0 - 2 per model, when approximated from the normal distribution according to the traditional Chauvenet’s criterion [19]. A few outliers were clearly influential, as they were located at the end of the scale far from the mean value of *x* (Additional file 1) and thus they affected slopes of the resulted regression models more than other values one at a time. Residuals were normally distributed in all cases except skin melanoma and rectal cancer, which also had the highest number of outliers (Additional file 1). The goodness-of-fit was thus low for the skin melanoma and rectal cancer models.The explanatory power was highest for the prostate cancer model (Figure 2) and lowest for the lung cancer model (Figure 3). Most models failed to estimate the incidences in individual municipalities in northern Finland, which was most probably due to a less comprehensive geographical coverage of the study area. Municipalities in northern Finland are large and sparsely populated and therefore the observed disease incidences do not represent the settlement concentrations of the municipalities as well as in middle and southern Finland. The small municipalities in Åland Archipelago and in the main island of Åland in southwestern Finland, however, were responsible for the residual extremums in the models. Standardized residuals > |4| were all connected to the municipalities in Åland as follows: lung and breast cancer in Sottunga (7.36 and 6.49, respectively), skin melanoma in Lumparland (7.62) and Sottunga (4.29), rectal cancer in Kökar (4.27) and Sottunga (-4.71), and colon cancer in Sund (4.25). Underestimated cancer incidences (high residuals) were much more common than overestimated incidences (low residuals).

**Table 1:** Explanatory power of linear regressions

**Cancer type**	***r***	***r***^**2**^	***r***^**2 **^adjusted*	***r***^**2 **^**adjusted**^**+**^
Prostate	0.814	0.663	0.638 (22)	0.579 (10)
Breast	0.788	0.621	0.589 (25)	0.552 (17)
Colon	0.764	0.584	0.559 (18)	0.479 (6)
Rectal	0.681	0.463	0.428 (20)	0.302 (6)
Leukemia	0.616	0.380	0.331 (23)	0.242 (11)
Stomach	0.623	0.388	0.356 (16)	0.216 (4)
Melanoma	0.580	0.337	0.316 (10)	0.130 (1)
Lung	0.574	0.329	0.289 (18)	0.129 (4)

*Based on the alpha level (*p*-value <0.05) stopping criterion; the number of explanatory vectors included in brackets.

^+^Based on the double stopping criterion; the number of explanatory vectors included in brackets. See text for a more detailed description of the method.

**Figure 4:**
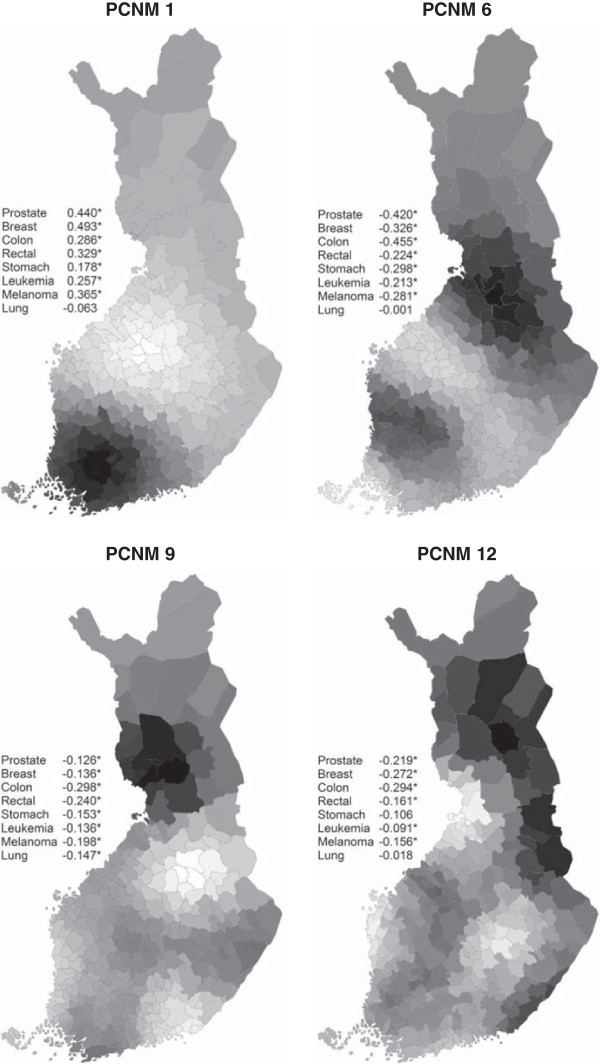
**Single spatial vectors which explained the incidences of different cancer types most.** Areas with high positive and high negative site scores in the PCNM vector have been denoted by dark and light colors, respectively. The number after the name of the cancer type informs the correlation coefficient (Pearson’s *r*) for the relationship between the incidence and the vector in question.

The spatial variation in prostate, breast, and colon cancer incidences were especially well explained by the vectors. The PCNM vectors 1, 6, 9, and 12 had the greatest explanatory power on the variation of cancer incidences in general and their standardized coefficients were > |0.2| in six, seven, eight, and six cases, respectively (Figure 4, Additional file 2). Although the incidences of nearly all cancer types were captured by the same spatial vectors with different combinations, some variation was also captured by a few vectors that were cancer type-specific. Prostate, colon, and stomach cancers were mainly associated with large-scale vectors, whereas breast cancer was related to large- and small-scale vectors, leukemia to scales of all sizes, and lung cancer to vectors reflecting medium-sized scales (Additional files 1 and 2).

## Discussion

### What PCNM can bring to spatial epidemiology?

This analysis demonstrates the usability of PCNM for producing statistically valid models of cancer incidence distribution. In the models, PCNM vectors can be used as proxies for demographics and causative agents to deal with autocorrelation, multicollinearity, and confounding variables. This, however, does not mean that PCNM vectors can replace explicit causative agents, when modeling disease incidences. Rather, spatial modeling can act as a complementary method to causal modeling 1) to achieve a better understanding of the spatial structure of both the response and explanatory variables and 2) to assess the spatial importance of unmeasured and/or previously unknown explanatory factors.

Spatial structures are rarely simple enough to be captured by a single PCNM vector. Grouping the PCNM vectors by scale is the procedure to uncover underlying processes occurring at different spatial scales [5,9]. These vector combinations may help to interpret the results as the most evident spatial processes are more clearly separated from each other. Our example was carried out in Finland, where many reasons for the non-uniform spatiotemporal distribution of different cancer types are known due to the high quality and large number of registers containing information about diseases themselves, socio-demographics, and many health-related issues. Importantly, PCNM enables studying areas where the availability of information is restricted, and is applicable for assessing the spatial distribution of different diseases. Because the countries without comprehensive registers are often developing countries with inadequate health care, poor health conditions, and high prevalence of serious infectious diseases, benefits from this kind of epidemiological research can be very high. As sample locations can be randomly or systematically assigned, PCNM does not require a register as a data source, but (any kind of) findings which can be located on a map. In this case, a map refers to (any kind of) 2- or 3-dimensional area, such as country, city, and building. To maintain comparability between the corresponding PCNM variables, the locations have to remain the same through time.

PCNM also improves the cost-effectiveness of epidemiologic research, as primary models can be created on the basis of unsupervised spatial vectors before collecting data on factors affecting the outcome. These primary models can be utilized in first-line evaluations of the research impact. They also serve as excellent predecessors to more explicit research questions. In spatial epidemiology, the PCNM vectors can be used to identify possible causative agents of diseases. If the disease and the exposure obey the same pattern of spatial vectors, they have to be somehow connected. A connection itself is not a proof of causal relationship, but all previously unknown associations can be valuable pointers for further research. In addition to spatial similarities between diseases and their causative agents, areas where disease incidences do not follow modeled spatial patterns can be of interest. These “exceptions to the rule” -areas denote that an exposure specifically important to these areas is missing from the model. The technique known as variation partitioning can be coupled to PCNM to identify the proportions of explained spatial and non-spatial variation [20].

PCNM may help to model and visualize changes in disease distributions. The areas created on the basis of PCNM vectors are not predecided but data-driven and thus flexible. This is a drastic contrast to methods based on preappointed permanent sites, such as the method currently used to visualize spatiotemporal variation in cancer in Finland [21]. This does not mean that the latter methods are inadequate, but highlights the applicability of PCNM. PCNM also enables generating models that do not aim to forecast each sample site but larger areas simultaneously, which again improves cost-effectiveness. If certain sites are explained by the same spatial vectors the number of sample sites and/or sampling frequency can be lowered.

Although the most evident solution to model spatiotemporal variation is to deal with the temporal and spatial effects as separate factors, methods for modeling non-separate space-time variation are also available [22,23]. Especially, Bayesian statistics provides solutions to model the spatial and temporal effects simultaneously [22,24], as well as to associate disease incidences with their causative agents [25]. In PCNM, it is possible to separate time and space, or to include time as a dimension in the input matrix. The latter solution enables modeling of phenomena which are affected by variables with varying spatial patterns and/or temporal cycles. In the absence of replication, spatiotemporal structures can also be studied using PCNM variables by the analysis of variance technique [26]. In this study, we intentionally excluded temporal aspects of variation in cancer incidences because we expressly wanted to test PCNM in the context of spatial epidemiology and create easily interpretable vectors representing 2-dimensional space.

### Limitations of the present study and the PCNM method in general

Variation in the vector combinations partly explained the differences in explanatory power of the regression models for different cancer types. If the incidence is explained mainly by PCNM vectors reflecting large-scale variation (the first PCNM variables), as in the case of prostate cancer (Table 1, Additional file 2), the explanatory power of the resulted model is high *per se*. Each small-scale vector corresponding to variation in a highly restricted geographical area in turn is able to explain only a small part of the total variation and thus a combination of several small-scale vectors is needed to reach the explanatory power of one large-scale vector, as was the case with leukemia (Table 1, Additional file 2). In other words, If the true spatial variation of the dependent variable is low (e.g., lung cancer; Figure 3) the explanatory power of the PCNM variables will also be low. The last PCNM vectors typically model case-specific peculiarities and they are of very limited general interest.

Migration may distort the connections between disease incidences and sites. The spatial patterns of observed disease incidence do not necessarily reflect the spatial patterns of exposures that have initiated the development of the disease. Explaining the observed disease incidence on the basis of spatial patterns is unreliable and even unadvised if the dataset consists of individual-level data and has high migration rate. This is especially problematic in the case of diseases with slow progression rates. Moreover, if the spatial distribution is random rather than systematic it cannot be predicted on the basis of spatial vectors reflecting cyclic variation.

## Conclusions

PCNM may help to extend spatial epidemiology to new areas, improve the cost-effectiveness *via* primary models and aid in identifying previously unknown causative agents and predict future trends in disease distributions and incidences. The models created with eigenvectors can be valuable as such to deal with autocorrelation, multicollinearity, and confounding variables or as predecessors for more explicit research questions. PCNM will benefit especifically early phase explorative research. A large advantage of using PCNM is that it can create statistically valid reflectors of real predictors for disease incidence models with only little resources and background information.

## Competing interests

The authors declare that they have no competing interests.

## Authors’ contributions

AV led the design, analysis and interpretation of data and the drafting of the article, A-MT, KV-J and PRS made substantial contributions to the drafting of the article. All authors approved of the final version of the article.

## Supplementary Material

Additional file 1 Relationships between observed and predicted cancer incidences and standardized model residuals. Click here for file

Additional file 2 Standardized coefficients resulted in regression models on cancer incidences. Click here for file

## References

[B1] Ostfeld RS Glass GE Keesing F Spatial epidemiology: an emerging (or re-emerging) discipline TREE 2005 20 328 336 16701389 10.1016/j.tree.2005.03.009

[B2] Tu Y-K Gilthorpe MS Statistical Thinking in Epidemiology 2012 Boca Raton, FL: CRC Press/Taylor & Francis Group

[B3] Woolston A Tu Y-K Gilthorpe MS Baxter PD Measuring the impact of collinearity in epidemiological research IJSP 2013 2 1 11

[B4] Hernán MA Hernández-Díaz S Werler MM Mitchell AA Causal knowledge as a prerequisite for confounding evaluation: an application to birth defects epidemiology Am J Epidemiol 2002 155 176 184 10.1093/aje/155.2.176 11790682

[B5] Borcard D Legendre P All-scale spatial analysis of ecological data by means of principal coordinates of neighbour matrices Ecol Model 2002 153 51 68 10.1016/S0304-3800(01)00501-4

[B6] Dray S Legendre P Peres-Neto PR Spatial modeling: a comprehensive framework for principal coordinate analysis of neighbour matrices (PCNM) Ecol Model 2006 196 483 493 10.1016/j.ecolmodel.2006.02.015

[B7] Moran PAP Notes on continuous stochastic phenomena Biometrika 1950 37 17 23 10.1093/biomet/37.1-2.17 15420245

[B8] Bivand RS Pebesma E Gómez-Rubio V Applied Spatial Data Analysis with R 2013 New York, NY: Springer

[B9] Borcard D Legendre P Avois-Jacquet C Tuomisto H Dissecting the spatial structure of ecological data at multiple scales Ecology 2004 85 1826 1832 10.1890/03-3111

[B10] Besag J York J Mollie A Bayesian image restoration, with two applications in spatial statistics AISM 1991 43 1 59 10.1007/BF00116466

[B11] Clayton D Kaldor J Empirical Bayes estimates of age-standardized relative risks for use in disease mapping Biometrics 1987 43 671 681 10.2307/2532003 3663823

[B12] Kamangar F Dores GM Anderson WF Patterns of cancer incidence, mortality, and prevalence across five continents: defining priorities to reduce cancer disparities in different geographic regions of the world J Clin Oncol 2006 24 2137 2150 10.1200/JCO.2005.05.2308 16682732

[B13] Teppo L Pukkala E Lehtonen M Data quality and quality control of a population-based cancer registry Acta Oncol 1994 33 365 369 10.3109/02841869409098430 8018367

[B14] Official Statistics of Finland (OSF) Migration 2012 http://www.stat.fi. Accessed October 2, 2013

[B15] Official Statistics of Finland (OSF) Population structure 2013 http://www.stat.fi. Accessed October 5, 2013

[B16] The R Foundation for Statistical Computing R version 2.11.1 2010 Vienna, Austria: The R Foundation http://www.r-project.org. Accessed November 28, 2013

[B17] Blanchet FG Legendre P Borcard D Forward selection of explanatory variables Ecology 2008 89 2623 2632 10.1890/07-0986.1 18831183

[B18] Gilbert B Bennett JR Partitioning variation in ecological communities: do the numbers add up? J Appl Ecol 2010 47 1071 1082 10.1111/j.1365-2664.2010.01861.x

[B19] Chauvenet W Manual of Spherical and Practical Astronomy 1863 Philadelphia, PA: J. B. Lippincott & Co.

[B20] Legendre P Borcard D Peres-Neto PR Analyzing beta diversity: partitioning the spatial variation of community composition data Ecol Monogr 2005 75 435 450 10.1890/05-0549

[B21] Pukkala E Söderman B Okeanov A Strom H Rahu M Hakulinen T Becker N Stabenow R Bjarnadottir K Stengrevics A Gurevicius R Glattre E Zatonski W Men T Barlow L Cancer atlas of Northern Europe Cancer Soc Finland Publ 2001 62 1 69

[B22] Knorr-Held L Bayesian modeling of inseparable space-time variation in disease risk Stat Med 2000 19 2555 2567 10.1002/1097-0258(20000915/30)19:17/18<2555::AID-SIM587>3.0.CO;2-# 10960871

[B23] Schrödle B Held L Spatio-temporal disease mapping using INLA Environmetrics 2011 22 725 734 10.1002/env.1065

[B24] Manda SOM Feltbower RG Gilthorpe MS Investigating spatio-temporal similarities in the epidemiology of childhood leukaemia and diabetes Eur J Epidemiol 2009 24 743 752 10.1007/s10654-009-9391-2 19784553

[B25] Downing A Forman D Gilthorpe MS Edwards KL Manda SOM Joint disease mapping using six cancers in the Yorkshire region of England Int J Health Geogr 2008 7 41 10.1186/1476-072X-7-41 18662387 PMC2515288

[B26] Legendre P De Cáceres M Borcard D Community surveys through space and time: testing the space-time interaction in the absence of replication Ecology 2010 91 262 272 10.1890/09-0199.1 20380215

