# Supplementary material for: From spatial ecology to spatial epidemiology: modeling spatial distributions of different cancer types with principal coordinates of neighbor matrices
Source: Emerg Themes Epidemiol. 2014 Aug 8;11:11. doi: 10.1186/1742-7622-11-11 (PMC4131804; doi:10.1186/1742-7622-11-11)
Supplement: Additional file 1 — Relationships between observed and predicted cancer incidences and standardized model residuals. [file 1742-7622-11-11-S1.pdf]

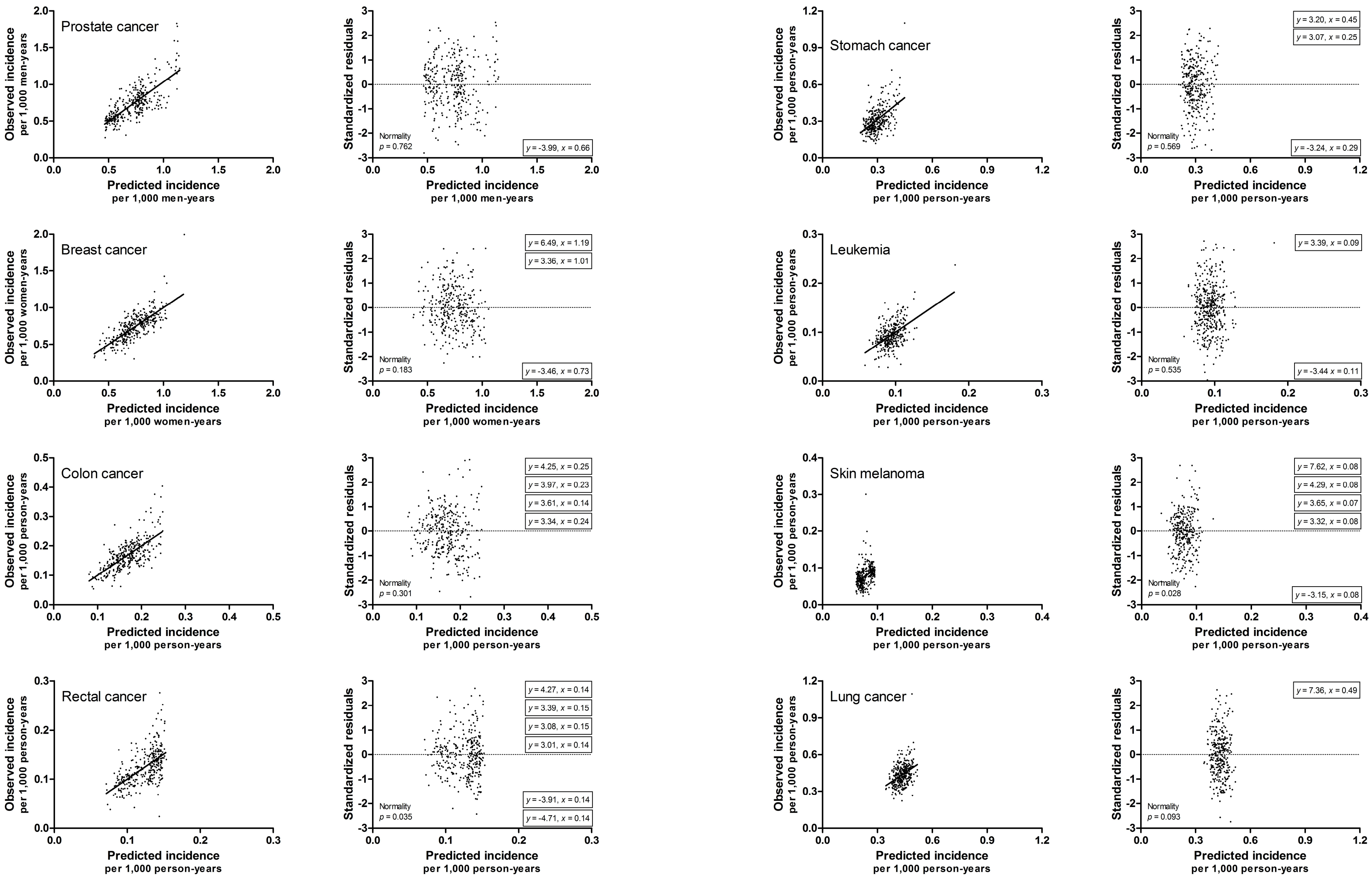

Additional file 1 Relationships between observed and predicted cancer incidences and relationships between standardized model residuals and predicted incidences. The number of explanatory PCNM vectors in the models was chosen on the basis of the double stopping criterion. Residuals >|3| are set out in the residual figures. Normality informs *p*-value for the Kolmogorov-Smirnov test. Note the varying scales of *y*- and *x*-axes.
