# Supplementary material for: From spatial ecology to spatial epidemiology: modeling spatial distributions of different cancer types with principal coordinates of neighbor matrices
Source: Emerg Themes Epidemiol. 2014 Aug 8;11:11. doi: 10.1186/1742-7622-11-11 (PMC4131804; doi:10.1186/1742-7622-11-11)
Supplement: Additional file 2 — Standardized coefficients resulted in regression models on cancer incidences. [file 1742-7622-11-11-S2.pdf]

Additional file 2 Standardized coefficients resulted in stepwise regression models on cancer incidences

Vector no.	Prostate	Breast	Colon	Rectal	Stomach	Leukemia	Melanoma	Lung
1	**0.440**	**0.493**	**0.286**	**0.329**	**0.178**	**0.257**	**0.365**	–0.063
2	**0.240**	**0.076**	–0.003	**–0.134**	**0.271**	0.007	–0.074	–0.057
3	**–0.178**	**–0.073**	**–0.078**	**–0.208**	–0.051	0.055	–0.011	**0.148**
4	0.020	–0.043	**–0.107**	**–0.175**	**–0.123**	–0.056	–0.003	**–0.211**
5	**0.123**	**0.080**	**0.114**	0.023	**0.126**	0.028	–0.045	0.037
6	**–0.420**	**–0.326**	**–0.455**	**–0.224**	**–0.298**	**–0.213**	**–0.281**	–0.001
7	**0.140**	**–0.120**	**0.109**	**0.095**	0.074	0.081	–0.026	–0.033
8	**–0.161**	–0.043	–0.038	**0.120**	0.016	0.010	0.023	–0.075
9	**–0.126**	**–0.136**	**–0.298**	**–0.240**	**–0.153**	**–0.136**	**–0.198**	**–0.147**
10	**0.081**	0.043	0.035	0.000	–0.033	0.067	0.043	0.033
11	0.007	**–0.083**	–0.042	–0.029	**–0.099**	0.053	–0.082	0.017
12	**–0.219**	**–0.272**	**–0.294**	**–0.161**	–0.083	**–0.091**	**–0.156**	–0.018
13	–0.052	–0.045	–0.018	0.033	0.024	**–0.139**	**–0.102**	–0.087
14	**–0.075**	**–0.149**	–0.068	–0.040	–0.087	–0.050	–0.044	0.010
15	–0.056	**–0.097**	–0.044	0.035	0.072	**0.099**	–0.061	–0.022
16	–0.030	0.028	–0.008	–0.036	–0.042	–0.078	–0.087	–0.024
17	–0.047	–0.024	–0.066	–0.035	–0.023	–0.065	0.019	–0.013
18	0.001	0.033	0.038	0.000	–0.025	0.014	0.022	–0.025
19	0.007	0.024	–0.021	–0.007	–0.084	–0.064	0.059	–0.074
20	–0.009	–0.031	–0.016	**0.098**	**0.095**	0.004	–0.071	0.044
21	**0.084**	0.023	0.045	**0.089**	**0.118**	**0.109**	–0.011	0.071
22	**–0.071**	0.010	–0.044	–0.052	**–0.130**	**–0.103**	–0.001	**–0.219**
23	–0.030	–0.015	0.003	0.024	0.044	–0.044	–0.029	0.001
24	0.050	0.051	0.018	0.021	–0.018	0.010	0.052	–0.048
25	0.015	–0.052	0.035	**0.097**	0.006	–0.033	0.069	0.014
26	–0.042	0.026	–0.066	–0.028	–0.052	–0.060	0.024	–0.085
27	–0.040	–0.003	**–0.088**	–0.026	0.005	–0.004	–0.048	0.041
28	0.061	**0.084**	0.049	–0.015	–0.071	–0.058	0.027	–0.043
29	0.038	0.038	0.058	0.066	0.053	0.086	–0.013	0.052
30	–0.055	–0.043	**–0.092**	**–0.094**	**–0.107**	–0.009	–0.089	**–0.099**
31	–0.059	–0.056	–0.014	0.025	0.053	–0.021	–0.052	0.059
32	**–0.073**	–0.031	**–0.110**	–0.040	–0.053	–0.052	–0.043	–0.063
33	0.032	–0.039	–0.019	–0.011	0.003	0.040	–0.010	–0.029
34	0.027	0.055	–0.050	–0.021	–0.048	–0.013	–0.069	**–0.113**
35	–0.016	0.014	0.064	0.000	0.024	0.046	0.024	0.001
36	–0.038	0.054	–0.021	0.073	–0.027	–0.081	0.020	–0.060
37	–0.021	–0.029	–0.054	0.019	0.039	–0.036	–0.084	0.005
38	**0.091**	–0.022	–0.006	–0.037	–0.002	**0.098**	–0.008	–0.044
39	–0.044	–0.029	–0.055	–0.031	–0.078	–0.043	–0.040	**–0.108**
40	0.030	0.022	–0.005	0.060	0.037	0.027	0.031	–0.069
41	–0.035	–0.013	–0.036	–0.064	–0.068	–0.003	–0.021	0.015
42	–0.055	0.029	**–0.080**	–0.012	–0.017	0.012	–0.065	–0.032
43	0.034	0.037	**0.103**	0.068	**0.139**	–0.067	–0.027	**0.130**
44	0.025	0.009	0.026	–0.024	–0.021	0.005	–0.017	**–0.113**
45	–0.019	–0.002	0.016	0.048	–0.003	**0.091**	0.040	0.019
46	0.018	0.009	0.046	–0.025	0.037	–0.010	0.007	0.022
47	–0.012	0.058	–0.004	–0.024	0.008	0.054	0.004	0.022
48	0.012	–0.003	0.012	0.000	**0.103**	0.027	–0.025	**0.115**
49	–0.063	0.018	–0.056	–0.033	–0.039	–0.017	–0.013	–0.082
50	0.059	0.045	0.007	0.013	0.082	–0.005	0.003	–0.042
51	0.012	0.060	–0.033	–0.033	–0.031	0.031	0.014	–0.043
52	0.025	–0.049	0.014	0.042	0.044	–0.036	–0.025	0.009
53	–0.025	–0.034	–0.067	–0.068	**–0.180**	**–0.093**	–0.086	**–0.128**
54	0.007	**0.073**	0.018	–0.071	0.011	0.022	–0.038	–0.004
55	–0.015	0.063	–0.028	–0.016	–0.022	**–0.095**	–0.024	–0.072
56	0.023	–0.048	–0.042	–0.049	–0.042	–0.065	0.006	–0.041
57	0.012	0.027	–0.018	0.010	0.059	0.012	0.038	0.046
58	**–0.072**	–0.035	**–0.079**	–0.034	–0.005	–0.010	–0.045	–0.019
59	–0.013	–0.063	0.018	–0.022	–0.010	0.023	–0.017	–0.016
60	–0.014	0.012	–0.061	**–0.087**	–0.029	**0.102**	0.036	0.001
61	–0.011	–0.047	0.024	0.036	0.035	0.003	–0.001	0.048
62	0.051	0.015	–0.022	–0.037	0.059	0.045	0.041	0.080
63	0.007	–0.029	0.015	–0.026	0.003	–0.064	–0.041	–0.032
64	–0.033	0.008	0.003	–0.002	–0.061	–0.035	–0.013	–0.019

65	–0.036	–0.042	–0.050	–0.047	0.025	–0.069	–0.006	0.011
66	–0.009	0.035	–0.001	–0.051	–0.001	0.063	–0.014	0.026
67	–0.017	–0.036	–0.021	–0.046	–0.013	–0.039	–0.051	**–0.125**
68	0.002	–0.001	–0.011	0.001	–0.080	0.022	0.055	**–0.099**
69	–0.027	0.025	0.008	0.020	0.046	–0.053	–0.074	0.015
70	0.012	0.019	0.017	0.033	–0.016	0.078	–0.059	0.000
71	–0.048	0.044	0.028	–0.008	–0.050	–0.021	–0.083	–0.012
72	–0.021	–0.027	–0.016	–0.029	–0.029	–0.070	–0.002	–0.027
73	0.021	–0.006	0.035	0.076	0.020	0.052	0.016	–0.007
74	0.019	–0.007	0.039	0.069	–0.015	0.045	0.018	0.035
75	0.017	0.052	0.056	–0.014	0.053	0.089	0.022	**0.094**
76	–0.009	0.030	0.039	–0.001	0.016	**0.135**	–0.014	0.016
77	0.012	–0.001	–0.001	–0.009	–0.016	0.012	0.034	–0.026
78	**–0.071**	**–0.105**	0.004	–0.012	0.002	0.014	–0.026	–0.028
79	–0.045	–0.070	0.004	–0.012	0.000	–0.017	0.006	–0.033
80	–0.013	–0.022	–0.005	–0.051	0.034	–0.035	**–0.103**	–0.002
81	–0.040	0.036	0.000	0.025	–0.045	–0.037	0.009	–0.072
82	0.047	–0.032	–0.039	0.062	0.003	0.034	–0.024	0.021
83	0.008	0.042	0.001	0.030	0.021	0.041	–0.068	0.039
84	0.062	0.053	0.057	0.002	–0.003	0.010	–0.013	0.005
85	0.035	0.012	0.012	–0.028	–0.059	–0.036	0.000	–0.015
86	–0.016	–0.002	0.006	–0.023	0.039	0.078	–0.081	0.052
87	–0.034	0.018	0.064	**0.095**	0.005	–0.003	0.086	0.008
88	0.030	–0.009	–0.050	0.036	–0.033	**–0.096**	0.030	–0.063
89	0.053	**0.078**	0.066	**0.106**	0.072	0.059	–0.050	0.082
90	0.001	0.035	–0.004	–0.047	–0.002	–0.055	0.023	–0.052
91	0.012	0.044	0.022	0.057	–0.014	0.038	–0.001	–0.013
92	0.018	–0.036	–0.058	0.039	0.011	–0.038	0.015	–0.001
93	**0.090**	0.052	0.047	0.052	0.014	**0.103**	0.015	0.045
94	0.011	0.028	0.005	–0.037	0.008	0.047	–0.023	–0.047
95	0.001	0.029	0.031	0.002	–0.024	–0.022	–0.028	0.037
96	–0.024	–0.048	**–0.083**	**0.109**	–0.045	–0.030	–0.035	–0.084
97	–0.019	–0.001	–0.031	–0.032	–0.076	–0.080	–0.017	**–0.161**
98	–0.042	–0.015	0.022	0.057	–0.003	0.011	–0.007	0.035
99	–0.031	0.067	–0.039	0.006	–0.027	–0.055	0.017	–0.048
100	–0.023	–0.058	0.021	–0.004	–0.004	0.033	–0.022	–0.017
101	0.005	0.057	–0.003	–0.054	0.000	0.037	0.075	0.036
102	–0.010	–0.015	–0.021	0.025	0.023	–0.012	**–0.092**	0.003
103	–0.037	–0.041	–0.003	0.016	0.025	0.023	–0.009	0.047
104	0.008	0.004	0.045	0.001	0.025	0.035	–0.011	–0.033
105	–0.060	**–0.086**	–0.042	–0.082	–0.005	–0.039	–0.051	0.013
106	–0.050	0.015	–0.019	0.021	–0.037	–0.036	0.006	–0.038
107	–0.027	–0.016	–0.034	–0.016	0.015	–0.003	–0.001	–0.001
108	–0.032	–0.066	0.018	–0.023	0.022	–0.005	0.010	0.027
109	–0.031	0.004	–0.007	–0.039	0.020	–0.001	0.056	0.017
110	–0.029	**0.089**	–0.023	**–0.100**	–0.059	0.057	0.021	0.034
111	–0.002	–0.006	–0.070	–0.017	–0.023	0.024	0.004	0.068
112	0.018	–0.051	0.020	0.049	0.014	0.014	–0.036	–0.036
113	–0.041	0.040	–0.010	–0.079	–0.044	0.048	–0.003	0.008
114	–0.039	**–0.122**	–0.055	0.032	–0.045	0.078	–0.078	–0.052
115	0.030	–0.068	0.060	0.043	**0.092**	0.082	0.051	0.028
116	–0.035	–0.040	0.021	–0.039	–0.060	–0.063	0.027	0.026
117	–0.048	–0.007	–0.032	–0.057	–0.003	0.051	–0.036	–0.004
118	–0.016	0.045	–0.008	0.022	–0.082	0.000	–0.037	–0.018
119	–0.017	–0.044	–0.068	–0.002	–0.022	**–0.113**	0.001	–0.024
120	**0.088**	0.032	0.000	**–0.098**	–0.014	0.054	0.050	–0.061
121	0.016	–0.001	0.018	0.059	0.060	**0.136**	–0.040	0.022
122	–0.040	0.002	–0.034	–0.018	–0.017	0.008	–0.056	–0.070
123	0.023	0.070	0.031	0.030	**0.091**	0.014	0.003	0.070
124	0.010	**0.084**	0.012	0.007	–0.052	0.000	–0.007	0.011
125	–0.001	0.010	–0.044	–0.056	–0.007	–0.005	0.020	0.024
126	**0.070**	0.031	0.018	0.001	0.062	–0.052	0.058	0.070
127	–0.033	–0.017	–0.007	–0.021	0.050	0.028	0.019	0.039
128	0.008	–0.014	0.046	0.020	–0.018	–0.059	0.063	–0.004
129	–0.020	–0.016	–0.052	–0.041	0.022	0.043	0.037	**–0.106**
130	–0.006	–0.021	0.026	–0.022	0.062	–0.033	0.007	0.016
131	–0.024	**–0.072**	0.035	0.076	0.021	–0.002	–0.007	0.057
132	0.044	0.027	0.000	0.042	0.041	–0.032	**–0.107**	0.032
133	**0.071**	0.057	**0.080**	–0.052	0.012	0.025	–0.018	0.015
134	0.027	0.008	–0.017	–0.074	–0.036	–0.030	–0.062	–0.051

135	–0.015	**0.076**	0.044	0.031	–0.038	–0.046	**0.107**	0.014
136	–0.035	–0.008	–0.064	–0.050	0.001	–0.021	–0.025	–0.015
137	0.016	–0.013	–0.003	–0.059	–0.014	0.052	0.079	0.011
138	0.000	0.013	–0.021	–0.003	0.016	–0.034	0.059	0.057
139	–0.050	**0.098**	–0.019	–0.042	–0.021	–0.040	–0.039	0.006
140	0.037	–0.006	–0.009	0.025	–0.025	0.072	0.017	0.040
141	0.036	–0.016	**0.085**	0.046	0.066	**0.108**	0.013	**0.138**
142	–0.066	–0.016	–0.018	–0.020	0.022	0.033	–0.086	–0.061
143	–0.013	–0.021	0.011	0.031	0.028	0.007	0.039	0.022
144	–0.019	0.013	–0.024	0.002	–0.003	**0.093**	0.027	0.008
145	–0.035	0.048	0.033	–0.033	–0.045	**–0.119**	0.086	0.014
146	0.015	**0.085**	0.015	–0.006	0.072	0.004	–0.051	0.089
147	0.012	**–0.093**	–0.009	0.028	0.032	0.060	**0.097**	–0.044
148	0.025	0.023	–0.036	–0.016	0.051	0.034	–0.020	0.072
149	–0.019	0.033	–0.039	–0.065	0.018	0.011	–0.023	–0.021
150	–0.022	–0.013	–0.007	0.019	0.012	–0.017	–0.005	0.046
151	–0.042	0.049	**–0.074**	–0.046	–0.083	0.013	0.024	0.033
152	0.035	0.011	0.016	–0.029	0.078	0.051	0.024	**0.094**
153	–0.014	–0.047	–0.041	0.011	0.040	0.033	–0.057	0.026
154	0.005	0.000	–0.042	–0.023	–0.066	–0.071	–0.058	–0.015
155	–0.025	–0.005	–0.019	–0.014	0.005	–0.007	0.086	0.027
156	**–0.070**	–0.032	–0.009	–0.018	–0.029	0.013	–0.030	–0.063
157	–0.032	–0.013	–0.003	**–0.106**	–0.019	0.028	0.017	0.023
158	0.031	0.017	0.017	–0.064	0.009	0.067	0.033	0.048
159	–0.010	–0.026	0.012	0.005	–0.030	–0.024	–0.022	–0.046
160	0.010	–0.024	–0.019	–0.002	–0.031	–0.013	0.000	–0.020
161	0.010	0.009	–0.045	0.001	0.027	–0.033	–0.044	–0.044
162	0.039	**0.071**	–0.032	0.029	0.010	**0.179**	–0.055	0.066
163	–0.012	–0.001	–0.036	–0.030	–0.025	–0.030	–0.017	–0.082
164	–0.021	–0.001	0.020	0.025	–0.006	**0.093**	–0.002	–0.037
165	–0.058	–0.046	–0.017	–0.019	–0.015	–0.036	–0.015	0.006

Coefficients for vectors having *p*-value <0.05 are bolded for emphasis.
